# The relationship between parental reflective functioning, attachment style, parental competence, and stress

**DOI:** 10.1192/j.eurpsy.2022.1781

**Published:** 2022-09-01

**Authors:** B. Szabó, M. Miklósi, J. Futó

**Affiliations:** 1 Eötvös Loránd University, Department Of Developmental And Clinical Child Psychology, Budapest, Hungary; 2 ötvös Loránd University, Department Of Developmental And Clinical Child Psychology, Budapest, Hungary

**Keywords:** Stress, attachment, mentalization, parenting

## Abstract

**Introduction:**

Previous studies indicated, that mentalization mediates the link between adult attachment and stress, however, this relationship was not tested before among non-clinical parents of children aged between 12 and 18 years.

**Objectives:**

The aim of this study was to explore the relationship between parental reflective functioning, attachment style, perceived parental sense of competence, and stress among parents.

**Methods:**

After providing written consent, 186 non-clinical mothers completed a questionnaire packet that included a demographic form, The Parental Reflective Functioning Questionnaire - Adolescent version, the Attachment Style Questionnaire, the Parental Sense of Competence Scale, and the Perceived Stress Scale. A moderated mediation analysis with parental sense of competence as a dependent variable, mother’s attachment style as an independent variable, certainty about mental states hypermentalization subscale as a mediator, and stress as a moderator was conducted.

**Results:**

In the moderated mediation analysis, the direct effect of the attachment style on the parental sense of competence in the case of preoccupied attachment style was significant (*p* < .001). The interaction term of the hypermentalization subscale by perceived stress was also significant in the case of low level (*w* = -1.57, *p* < .001) and high level of perceived stress (*w*= 1.21, *p* = .049) among mothers with a preoccupied attachment style.

**Conclusions:**

These findings suggest that the preoccupied attachment style is related to the parental sense of competence through certainty about mental states hypermentalization in case of low level and high level of perceived stress, so mentalization-based interventions are warranted.

**Disclosure:**

No significant relationships.

